# Peptides derived from CXCL8 based on *in silico* analysis inhibit CXCL8 interactions with its receptor CXCR1

**DOI:** 10.1038/srep18638

**Published:** 2015-12-22

**Authors:** Shinn-Jong Jiang, Je-Wen Liou, Chun-Chun Chang, Yi Chung, Lee-Fong Lin, Hao-Jen Hsu

**Affiliations:** 1 Department of Biochemistry, School of Medicine, Tzu Chi University, Hualien 97004, Taiwan; 2 Institute of Medical Sciences, Tzu Chi University, Hualien 97004, Taiwan; 3 Department of Laboratory Medicine, Tzu Chi Medical Center, Hualien 97004, Taiwan; 4 Department of Life Sciences, Tzu Chi University, Hualien 97004, Taiwan

## Abstract

Chemokine CXCL8 is crucial for regulation of inflammatory and immune responses via activating its cognate receptor CXCR1. In this study, molecular docking and binding free energy calculations were combined to predict the initial binding event of CXCL8 to CXCR1 for peptide drug design. The simulations reveal that in the initial binding, the N-loop of CXCL8 interacts with the N-terminus of CXCR1, which is dominated by electrostatic interactions. The derived peptides from the binding region of CXCL8 are synthesized for further confirmation. Surface plasmon resonance analyses indicate that the CXCL8 derived peptide with 14 residues is able to bind to the receptor CXCR1 derived peptide with equilibrium K_D_ of 252 μM while the peptide encompassing a CXCL8 K15A mutation hardly binds to CXCR1 derived peptide (K_D_ = 1553 μM). The cell experiments show that the designed peptide inhibits CXCL8-induced and LPS-activated monocytes adhesion and transmigration. However, when the peptides were mutated on two lysine residues (K15 and K20), the inhibition effects were greatly reduced indicating these two amino acids are key residues for the initial binding of CXCL8 to CXCR1. This study demonstrates that *in silico* prediction based functional peptide design can be effective for developing anti-inflammation drugs.

Excessive or prolonged leukocyte related inflammation generally leads to tissue destruction, which highlights the importance of properly controlling this inflammatory process. The inflammatory response is mediated by complex interactions between leukocytes and vascular endothelium. Activation of endothelium at the inflammatory sites causes leukocytes to transmigrate into the sub-endothelial space^1^. Chemokines mediate a wide range of biological functions via recruiting leukocytes to the site of injury and infection to organogenesis, wound healing, metastasis, and angiogenesis^2^,^3^,^4^,^5^. Chemokines are small signaling proteins that control tissue functions, including cell recruitment and activation under homeostatic or inflammatory conditions by binding and activating the G protein coupled receptors (GPCR) on the cell surface^5^. In humans, the chemokine CXCL8 (also known as interleukin-8 or IL-8) performs its function by activating its cognate receptors, CXCR1 and CXCR2^6^,^7^. Because CXCL8 binding to its receptors can increase tumor growth by promoting angiogenesis, CXCR1 has been identified as a target for blocking the formation of breast cancer stem cells and malignant melanoma that drive tumor growth and metastasis^8^,^9^. Thus, understanding CXCL8–CXCR1 interactions should greatly facilitate the development of strategies for preventing chronic diseases caused by prolonged inflammation.

The interactions between CXCL8 and CXCR1 have been largely studied by residue-based mutational analyses and NMR experiments. These studies have identified that the charge–charge interaction is critical for the binding of CXCL8 to CXCR1^10^,^11^,^12^. The ELR motif near the N-terminus (residues 4–6) and the N-terminal loop (N-loop) of CXCL8 have been implicated in the interactions with CXCR1^10^,^12^. Mutagenesis studies have also demonstrated that charged residues near the third and fourth extracellular loops (EC loops) of CXCR1 are crucial for these interactions^11^,^12^,^13^. Based on these studies, a mechanism by which CXCL8 and CXCR1 interact has been proposed as occurring in a two-sites multistep process^12^,^14^,^15^,^16^,^17^,^18^,^19^. The initial step corresponds to the recognition of the N-loop of CXCL8 to the N-terminal domain of CXCR1, which is driven predominantly by electrostatic interactions. The second step is the orientation change of CXCL8, caused by hydrophobic interactions, to allow the N-terminal ELR motif of CXCL8 to move closer toward the extracellular loops (EC loops) of CXCR1^16^,^19^,^20^. Finally, the ELR motif of CXCL8 binds to the EC loops of CXCR1 through electrostatic interactions (Site II binding), triggering conformational changes of CXCR1 that result in downstream signal transduction.

In past decades, peptides have been developed for regulating physiological processes or used therapeutically in diverse areas such as neurology, endocrinology, and haematology^21^. More recently, protein-capture peptides have also been widely used in protein detection, immobilization and assist the development of *in vitro* diagnostic chips^22^,^23^. CXCL8, because of its involvement in several cancers, has been suggested as a diagnostic marker or promising target for drug discovery^8^,^9^,^24^,^25^. Some CXCL8-binding peptides have been proposed to inhibit CXCL8 binding to human neutrophils^26^,^27^. In addition, a peptide derived from two short sequence motifs of the N-terminus of CXCR1 linked by a general sequence was verified as high affinity for CXCL8 binding^28^,^29^. However, to the best of our knowledge, no report exists regarding the peptide inhibition of CXCL8 binding to CXCR1.

We recently proposed that CXCL8 binding to CXCR1 is a multistep process, which is in accordance with previous experiments^19^. In the current study, we performed molecular docking to determine the preferable binding sites of CXCL8 to CXCR1, and peptide sequences predicted from the initial binding sites were selected to dock with CXCR1. The formed peptide–CXCR1 complex was then embedded in a POPC lipid bilayers for binding free energy calculations. Subsequently, peptides designed according to these calculations and their mutant counterparts were chemically synthesized for cellular assay and surface plasmon resonance (SPR) measurements for validating the genuine biological effect. The cellular assays were conducted to test the inhibitory effects of the designed peptides on CXCL8-induced immune response at the cellular level. In addition, because bacterial endotoxin lipopolysaccharides (LPS) could cause severe immune responses in humans, leading to severe sepsis or septic shock^30^,^31^, the inhibitory effects of the designed peptides on LPS-activated cellular inflammatory response were also examined. This study demonstrated an effective process for developing peptide drugs with inhibitory functions by using molecular docking predictions, binding free energy calculations, SPR measurements, and *in vitro* cellular assays.

## Results

### Construction and equilibration of the receptor CXCR1

Sequences comparisons show that the N- and C-terminal parts of human receptor CXCR1 and bovine rhodopsin (PDB: 1U19) are 18.7% identical and 41.8% similar. Following the protocol of our pervious study^19^, CXCR1 was constructed by combining the NMR experiment (PDB: 2LNL) and homology modeling of the N- and C-terminal parts. The equilibrated full-length CXCR1 structure was obtained by embedded into a POPC lipid bilayer for 100 ns MD simulations. The backbone RMSD values show stable fluctuations around 0.37 nm during the first 30 ns and gradually rising up to around 0.43 nm after 100 ns simulations (Fig. S1). The change of secondary structure elements during the 100 ns simulations indicated that the N-terminal part (residues 1–35), and extracellular parts (EC1: residues 102–108, EC2: residues 173–198, and EC3: residues 277–284) remain in their random coil and loop forms during the simulations (Fig. S2). The average structure of CXCR1 obtained based on PCA of the covariance matrix resulting from the last 30 ns trajectories showed that the long N-terminal and three extra cellular loops formed a groove for the ligand binding (Fig. S3).

### Molecular docking of full-length CXCL8 to CXCR1

**Figure 1:**
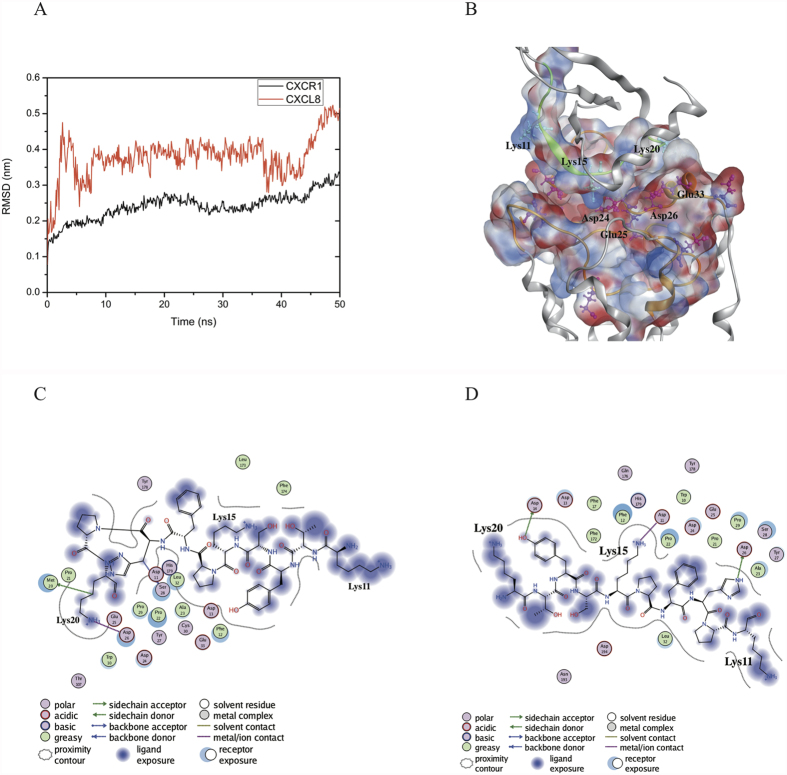
RMSD values and interaction maps of CXCL8 binding with CXCR1 during the initial MD simulations. (**A**) RMSD values for the backbone atoms of CXCL8 and CXCR1 during the first 50 ns MD trajectory. (**B**) The surface charge distribution of the average complex structure over the last 30 ns of the MD trajectory based on Poisson-Boltzmann equation, in which blue color corresponds to positive and red color to negative electrostatic potential. (**C**) The interaction map of CXCL8 initial binding to CXCR1. The cationic end of K20 of CXCL8 forms salt bridges with E25 and D26 of CXCR1. (**D**) The interaction map of average structure of the complex over the last 30 ns of the MD trajectory. The cationic end of K15 of CXCL8 forms a salt bridge with D11 of CXCR1; Y13 and H18 of CXCL8 form H-bonds with D14 and D26 of CXCR1.

In the initial stage the rigid-body docking algorithm ZDOCK generated a total of 54,000 CXCL8-CXCR1 complex structure poses. RDOCK was used to rerank and refine the poses from the clusters according to the ZDOCK results. The most preferable initial site for full-length CXCL8 binding to CXCR1 was selected for further 50 ns MD simulations. The RMSD values for the backbone atoms of CXCR1 gradually increased to 0.30 nm after 50 ns; in the case of CXCL8, these values fluctuated around 0.38 nm in the first 35 ns and then increased to 0.50 nm (Fig. 1A). The RMSF values for the C_α_ atoms of CXCR1 showed a high degree of fluctuations in the N-terminus (>0.40 nm), and EC1-2 (~0.27 nm), whereas the high fluctuations for CXCL8 were in the N-terminal loop and C-terminal helix (Fig. S4). The surface charge distribution of the average CXCL8–CXCR1 complex structure over the last 30 ns of the MD trajectory based on Poisson-Boltzmann equation is shown in Fig. 1B. During the initial binding stage, the end region of the N-loop (residues 14–20) of CXCL8 interacted with the groove region of the N-terminal domain (residues 21–27) of CXCR1. Positively charged residues of CXCL8, such as K11, K15, and K20, formed a positive electrostatic field near the N-loop, whereas negatively charged residues of CXCR1, such as D11, D14, D24, E25, and D26 formed a strong negative electrostatic field around the binding groove (Fig. 1B). The electrostatic interactions dominated the initial binding of CXCL8 with CXCR1. In the initial binding, the interaction maps show that the cationic end of K20 of CXCL8 forms salt bridges with the anionic ends of E25 and D26 of CXCR1 (Fig. 1C). The initial binding site is also consistent with previous NMR experiments of CXCR1 receptor fragment in complex with CXCL8^32^. However, for the interaction map of the average structure of the complex over the final 30 ns of the MD trajectory, the cationic end of K15 of CXCL8 forms a salt bridge with the anionic end of D11 of CXCR1, and Y13 and H18 of CXCL8 form H-bonds with D14 and D26 of CXCR1 (Fig. 1D). Hydrophobic residues of CXCR1 (F12, F17, P21, P22, P29, A23, L32, and F172) around the N-loop of CXCL8 during the MD simulations indicated that hydrophobic interactions may play a critical role in CXCL8–CXCR1 interactions. Therefore according to the docking results and refinement of MD simulations, the binding regions of ligand CXCL8 (residues 8–21, p_wt14) and receptor CXCR1 (residues 11–28, CXCR1p) were synthesized for the following confirmations of SPR detection and cellular assays. According to the surface charge distributions and interaction maps, three lysine residues (K11, K15, and K20) of the N-loop of CXCL8 may be the key residues at the initial binding stage.

### Binding free energy calculations for peptides derived from CXCL8

**Figure 2:**
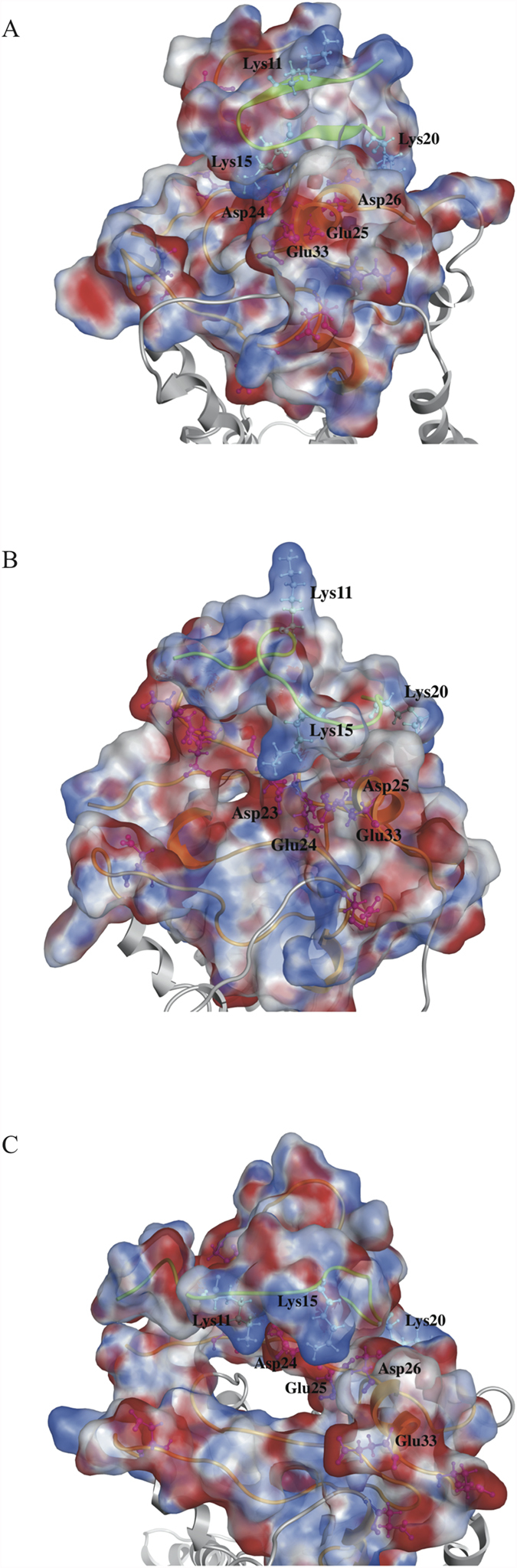
The surface charge distribution of the peptide-receptor complex structure based on Poisson-Boltzmann equation. The three peptides ((**A**) p_wt14 (**B**) p_wt16 (**C**) p_wt18) still bind to the groove of N-terminal domain of CXCR1 during the 50 ns MD simulations with positively charged lysine residues facing the negatively charged groove region of CXCR1 indicating that electrostatic interactions dominate the initial binding. Side chains of positively charged residues are represented as light blue color while that of negatively charged residues are represented as pink color.

**Figure 3:**
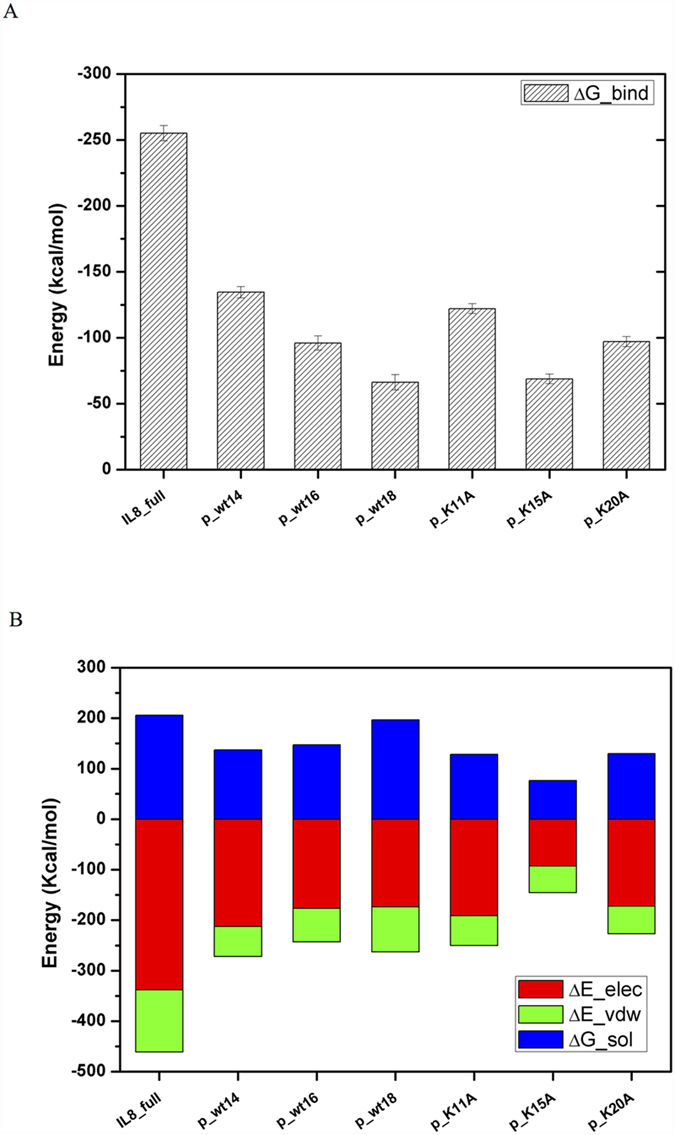
The MM/PBSA binding free energy calculations for various peptides of CXCL8 binding to CXCR1. (**A**) For wild peptides with different lengths (p_wt14, p_wt16 and p_wt18) and mutant peptides (p_K11A, p_K15A and p_K20A). (**B**) The detailed analysis of the components of binding free energies shows that electrostatic interactions dominate the binding (red color), followed by solvation free energies (blue color) and van der Waals (VDW) interactions (green color).

Figure 2 depicts the surface charge distributions for different peptides derived from CXCL8 binding to CXCR1 after 50 ns MD simulations. Peptides p_wt14, p_wt16, and p_wt18 still bind to the groove of N-terminal domain of CXCR1 during the 50 ns MD simulations with positively charged lysine residues facing the negatively charged groove region of CXCR1, indicating that electrostatic interactions dominate the initial binding (Fig. 2). From the surface charge distributions, although the three wild type peptides of CXCL8 with different lengths bind to the CXCR1 through electrostatic interactions, detailed binding free energies of various CXCL8 peptides to CXCR1 can aid to determine potential peptides for peptide drug development. The MM/PBSA binding free energy calculations for various wild type and mutant CXCL8 peptides binding to CXCR1 are summarized in Fig. 3 and Table S1. Peptide p_wt14 (−134.49 kcal/mol) had lower binding free energy than did the other two peptides (p_wt16, -96.06 kcal/mol and p_wt18, −66.24 kcal/mol). The binding free energy of CXCL8 derived peptide p_wt14 to the entire CXCR1 (−134.49 kcal/mol) is also lower than that of the other region of full-length CXCL8 excluding he peptide p_wt14 (−120.61 kcal/mol), meaning that p_wt14 dominates the binding to CXCR1 (Fig. 3A). Based on binding free energy calculations of the three wild type peptides to CXCR1, CXCR1 prefers binding with p_wt14 compared with binding with p_wt16 and p_wt18. The binding free energies of the three point mutant peptides (p_K11A, p_K15A, and p_K20A) to CXCR1 indicated that p_K15A has higher free energy than do p_K11A and p_K20A, meaning that the contribution of K15 to binding is more than that of the other two amino acids (K11 and K20) (Fig. 3A). Advanced analysis of the components of binding free energies revealed that electrostatic interactions dominated the initial binding, followed by solvation free energies and van der Waals (VDW) interactions (Fig. 3B). For p_wt18, as the peptide length extended, the solvation energy and VDW interactions increased while the electrostatic interactions decreased, implying that electrostatic interactions may not dominate the binding. Electrostatic interactions and solvation energy declined more for mutant peptide p_K15A than for the other two mutant peptides, indicating that K15 is the key residue in peptide binding to CXCR1 (Fig. 3B).

### SPR measurements for the interactions between CXCL8 and CXCR1 derived peptides

**Figure 4:**
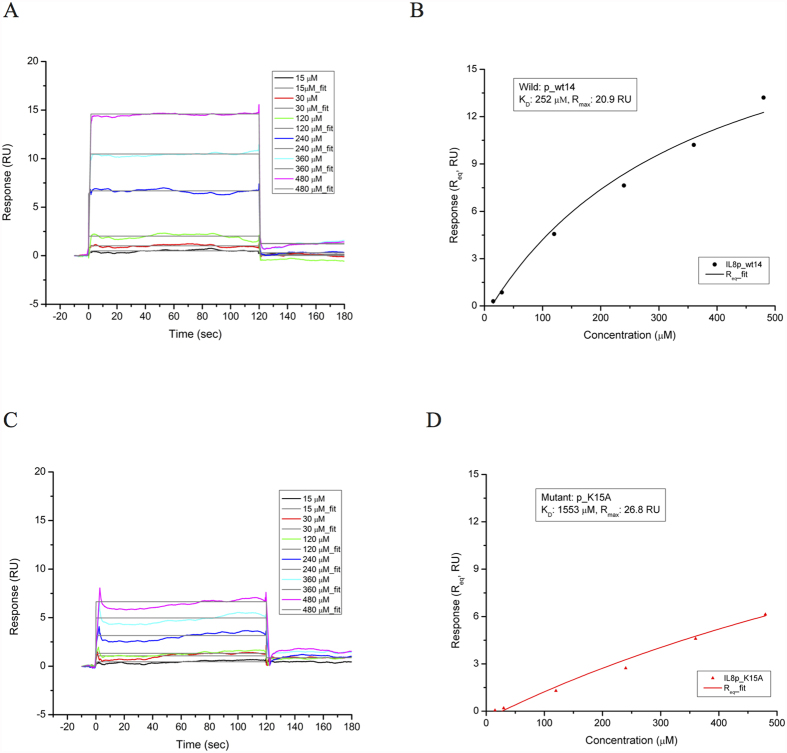
SPR analysis of the interaction between wild type and mutant ligand peptides and receptor peptide CXCR1p. (**A**) Receptor peptide CXCR1p was injected over the immobilized ligand peptide p_wt14. As the concentration of CXCR1p increased, the measured response for CXCR1p also increased, which indicated the concentration-dependent effect. (**B**) For steady-state interaction, a binding isotherm was created to determine the equilibrium K_D_ (252 μM) and R_max_ (20.9 RU) for CXCR1p binding to p_wt14. (**C**) Receptor peptide CXCR1p was injected over the immobilized ligand peptide p_K15A. The response units with time for various concentrations of CXCR1p binding to p_K15A immobilized on the CM5 chip are quite small. (**D**) For steady-state interaction, a binding isotherm was created to determine the equilibrium K_D_ (1553 μM) and R_max_ (26.8 RU) for CXCR1p binding to p_K15A.

Peptides p_wt14 and CXCR1p were synthesized for surface plasmon resonance (SPR) detection using a Biacore T200 instrument to determine whether p_wt14 would bind to the N-terminal region of CXCR1 (CXCR1p) and to assess how well it binds relative to the mutant peptide of CXCL8. SPR sensorgrams provided a positive change in response units (RUs), revealing that receptor peptide CXCR1p bound to the ligand peptide p_wt14 immobilized on the CM5 chip (Fig. 4A). As the CXCR1p concentration increased, the measured response for CXCR1p binding to p_wt14 also increased, indicating a concentration-dependent effect. After injection, CXCR1p bound to p_wt14 and the curves reached a plateau immediately in several seconds; furthermore, CXCR1p dissociated quickly during the rinsing of the chip with buffer (Fig. 4A). For steady-state interaction, a binding isotherm was created to determine the equilibrium dissociation constant K_D_ (approximately 252 μM) and R_max_ (approximately 20.9 RU) for CXCR1p binding to p_wt14 (Fig. 4B).

Based on the binding free energy calculations for the mutated peptides, mutant peptide p_K15A was selected for SPR measurement comparison with wild type peptide p_wt14. The RUs with time for various concentrations of receptor peptide CXCR1p binding to mutant ligand peptide p_K15A immobilized on the CM5 chip were quite small (<7.5 RU for 480 μM of p_K15A) (Fig. 4C) indicating that CXCR1p could scarcely bind to p_K15A, which is consistent with the binding free energy calculations (Fig. 3A). The kinetic analysis of binding isotherm showed that the equilibrium dissociation constant (K_D_) for CXCR1p to mutant p_K15A was much higher (K_D_ = 1553 μM, R_max_ = 26.8 RU) than that of wild p_wt14 (K_D_ = 252 μM) (Fig. 4B,D). In addition, the low binding affinity of mutant p_K15A revealed that residue K15 of CXCL8 is the key residue for p_wt14 binding to CXCR1p.

To confirm whether the binding affinity effect of these lysine residues can be attributed to side-chain amino groups rather than to drastic integral structure changes caused by lysine-to-alanine mutation, circular dichroism (CD) analysis of these peptides was performed (Fig. S5). The CD analysis indicated that all the peptides tested were random coil conformations, meaning that the lysine-to-alanine mutation only causes reduced electrostatic interactions and not the conformational changes of the peptides, which is consistent with the aforementioned binding free energy calculations (Fig. 3B).

### Peptides with different lengths for inhibiting monocyte adhesion to CXCL8-treated endothelial cells

**Figure 5:**
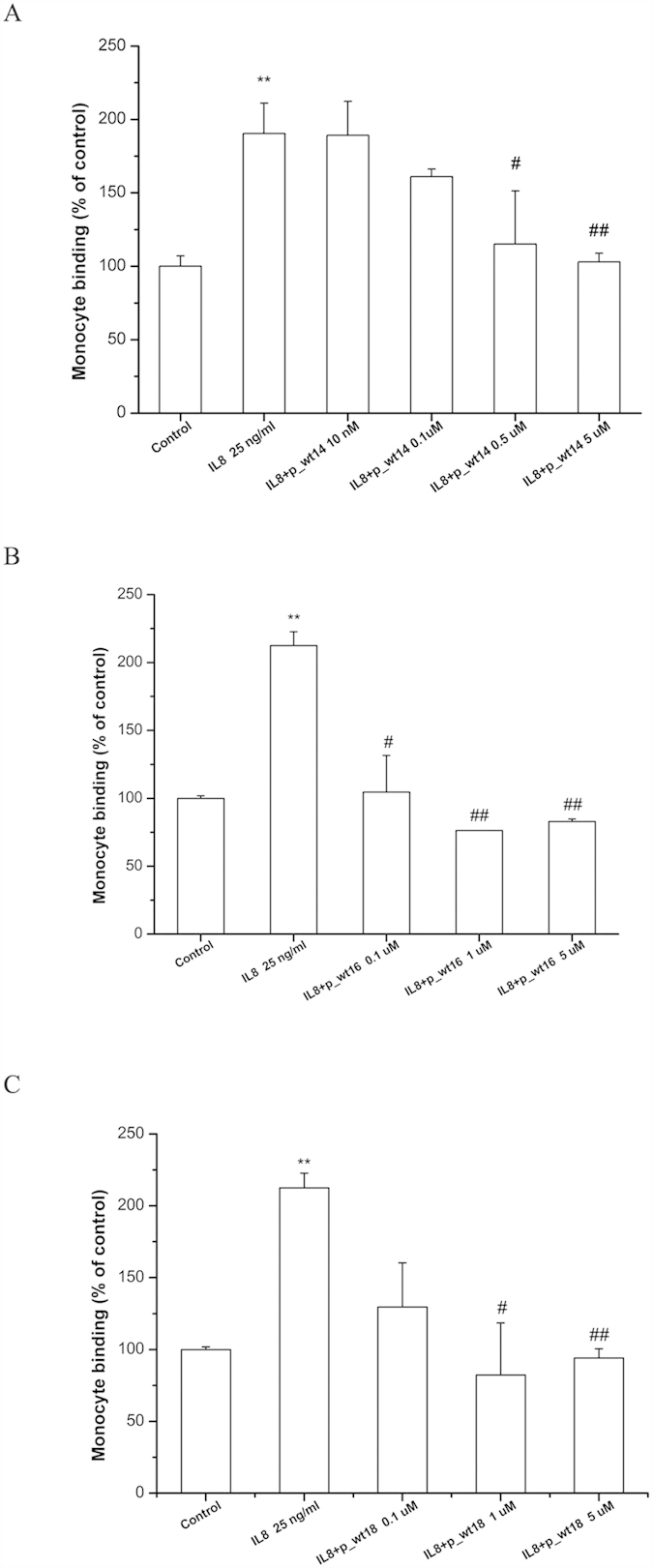
Peptides with various lengths inhibit CXCL8 induced monocyte adhesion to HMEC-1. (**A**) p_wt14 (**B**) p_wt16 (**C**) p_wt18. HMEC-1 was pretreated with various concentrations of the three peptides for one hour, and then stimulated with 25 ng/ml CXCL8 for 18 hours. Adhesion of fluorescent THP-1 cells was photographed by fluorescent microscopy and calculated. “Control” means that only the culture medium (without peptides) is incubated with cells. Values are mean ± SD from three independent experiments. (** *P* < 0.01) as compared to control; (^#^*P* < 0.05) and (^#^*P* < 0.01) as compared to cells stimulated with CXCL8 in the absence of the three peptides.

To confirm the predicted preferable peptides binding to CXCR1 by molecular docking, peptides with various lengths were synthesized for examining the adhesion of THP-1 cells to CXCL8 activated human microvascular endothelial cells (HMEC-1). As shown in Fig. 5A, the activation of HMEC-1 by CXCL8 caused a significant increase in the number of adhered monocytes on the endothelial cells. However, the pretreatment of HMEC-1 with p_wt14 dose-dependently reduced the number of THP-1 cells adhering to CXCL8-treated HMEC-1, with an almost 100% (*P* < 0.01) decrease, similar to the results for HMEC-1 pretreated with peptides p_wt16 and p_wt18 (Fig. 5B,C). All three peptides inhibited CXCL8-induced monocyte adhesion, indicating that the peptides all contained the initial binding region. Table S2 summarizes the IC50 values of the peptides used to inhibit CXCL8-induced monocyte adhesion to HMEC-1 cells. All the three peptides have the ability to inhibit CXCL8 binding to CXCR1. By comparing the binding free energies of these peptides, the peptide length with 14 amino acids (p_wt14) was found to be enough to suppress the binding of CXCL8 to CXCR1. To confirm whether the inhibition was caused by the predicted peptide (p_wt14), a nonrelated peptide with 11 amino acids (p_nr11, YSWGANDTDVF) was synthesized for monocyte binding assays (Fig. S6). Comparing Figs 5A and S6 revealed that though p_wt14 reduced CXCL8-induced monocyte binding, the nonrelated peptide (p_nr11) could not, demonstrating that, as predicted, only the predicted peptide (p_wt14) could bind the CXCR1.

**Figure 6:**
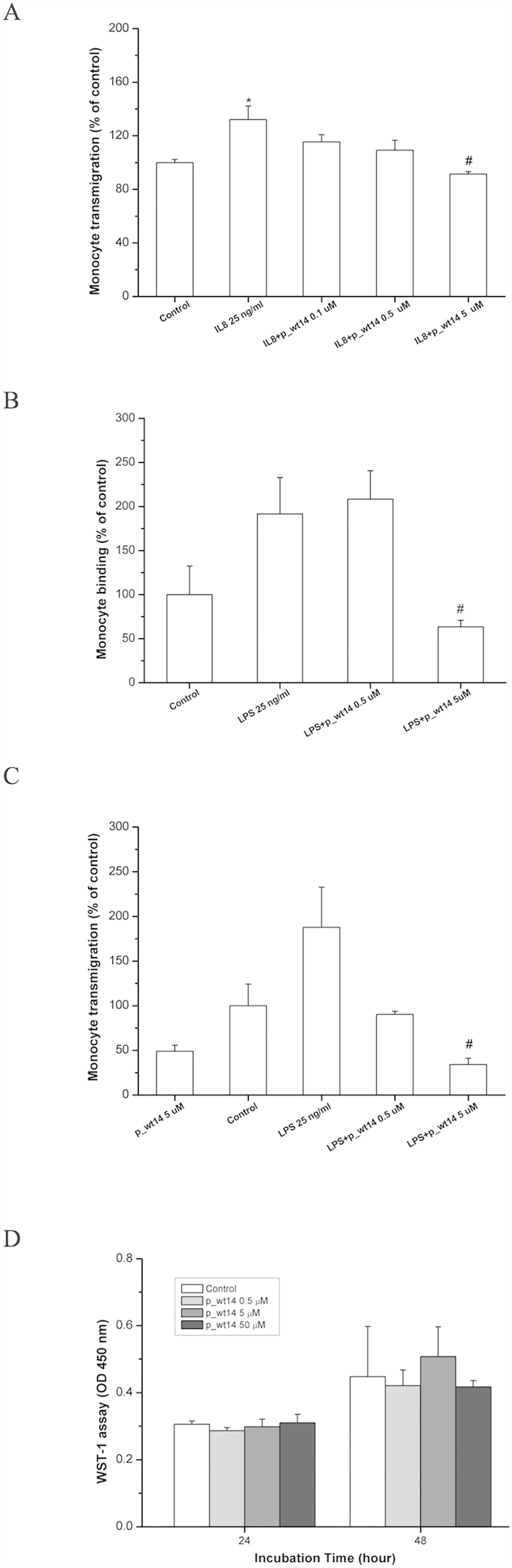
Predicted peptide p_wt14 inhibit CXCL8 and LPS induced monocytes adhesion and transmigration. (**A**) p_wt14 inhibits CXCL8 induced monocytes transmigration. HMEC-1 was pretreated with various concentrations of p_wt14 for one hour and then combined with 25 ng/ml CXCL8 for 18 hours of incubation, before the THP-1 cells were allowed to transmigrate through the HMEC-1 monolayer. The transendothelial migrated monocytes were measured and calculated by counting cells migrating to the bottom wells under microscopy. (**B**) p_wt14 inhibits LPS induced monocytes adhesion. (**C**) p_wt14 inhibits LPS induced transmigration. HMEC-1 was pretreated with various concentrations of p_wt14 for one hour and then combined with 25 ng/ml LPS for 18 hours of incubation, before the THP-1 were allowed to adhesion to HMEC-1 (**B**) or transmigrate through the HMEC-1 monolayer (**C**). Values are mean ± SD from three independent experiments. (*P < 0.05) as compared to control; (^#^*P* < 0.05) as compared to cells stimulated with CXCL8 or LPS in the absence of p_wt14. (**D**) p_wt14 does not induce the cytotoxic effect on endothelial cells. HMEC-1 was treated with various concentrations of p_wt14 in 96-well plate. After 24 and 48 hours of incubation, cell viability was evaluated using the colorimetric WST-1 assay. Data are the mean ± SD of triplicate determinations. “Control” means that only the culture medium (without peptides) is incubated with cells.

Furthermore, trans-well assays were used to study whether p_wt14 could inhibit CXCL8-induced monocyte transmigration. HMEC-1 cultured with CXCL8 exhibited a considerable increase in monocyte transmigration across the endothelium, compared with the migration in the absence of CXCL8. By contrast, the presence of p_wt14 reduced the ability of THP-1 cells to migrate across HMEC-1 to 100% (*P* < 0.01) in a dose-dependent manner (Fig. 6A). These results showed that peptide p_wt14 was able to inhibit CXCL8-induced monocyte transmigration. Bacterial antigen such as LPS binds with Toll-like receptors on the surface of endothelial cells to transduce the inflammatory signal into nucleus to regulate immune responses including CXCRs and CXCL8 expressions. To investigate how peptide p_wt14 affects the LPS-induced monocyte adhesion to endothelial cells, we examined the adhesion of THP-1 cells to LPS-activated HMEC-1. LPS-induced THP-1 cells adhere to HMEC-1 (twofold). Notably, THP-1 had an attenuated ability to bind to p_wt14-pretreated endothelial cells by 100% (*P* < 0.05) compared with LPS alone in a dose-dependent manner (Fig. 6B). Similarly, THP-1 transmigration was promoted under LPS stimulation (twofold). In addition, the number of transmigrated THP-1 cells on p_wt14 pretreated endothelial cells declined dose-dependently compared with the number after LPS exposure alone, exhibiting a 100% (*P* < 0.01) decrease (Fig. 6C). These results revealed that p_wt14 can inhibit LPS-induced monocyte adhesion and transmigration by blocking CXCR1 and CXCR2 activation. To determine whether the inhibitory effects of p_wt14 on monocyte adhesion and transmigration were due to only their cytotoxic effects on HMEC-1, endothelial cells were treated in the same manner as previously described. After 24 and 48 hours, HMEC-1 viability was evaluated using a WST-1 assay. As depicted in Fig. 6D, no significant cell viability difference was observed between the control and p_wt14-treated cells, suggesting that the inhibition of monocyte adhesion and transmigration by peptide p_wt14 was not due to endothelial cytotoxicity.

### Cell assays for confirmation of key residues on predicted peptide responsible for binding to CXCR1

**Figure 7:**
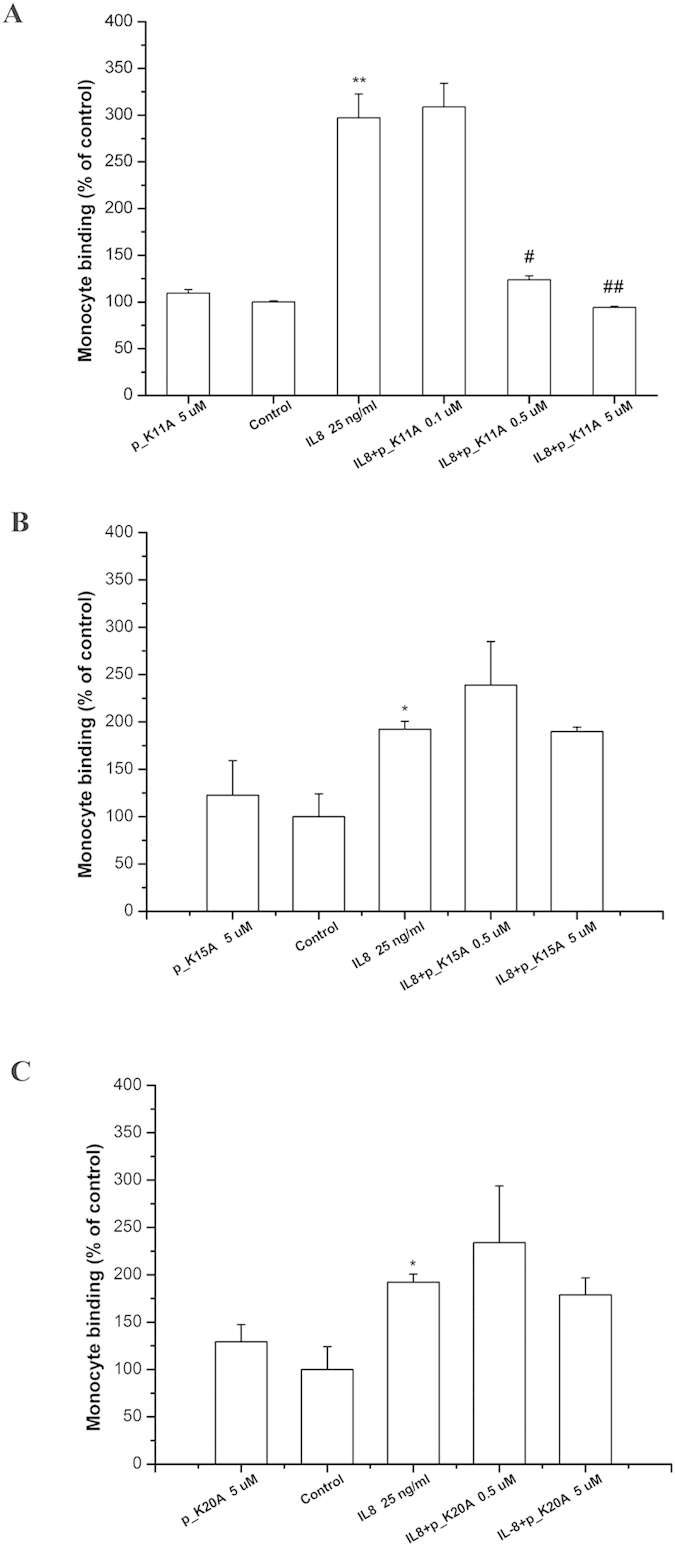
Comparison of various mutant peptides to inhibit CXCL8 induced monocyte adhesion to HMEC-1. (**A**) Mutant peptide p_K11A (**B**) Mutant peptide p_K15A (**C**) Mutant peptide p_K20A. HMEC-1 was pretreated with various concentrations of the mutant peptide for one hour, and then stimulated with 25 ng/ml CXCL8 for 18 hours. Adhesion of fluorescent THP-1 cells was photographed by fluorescent microscopy and calculated. “Control” means that only the culture medium (without peptides) is incubated with cells. Values are mean ± SD from three independent experiments. (**P < 0.01) as compared to control; (^#^*P* < 0.05) and (^#^*P* < 0.01) as compared to cells stimulated with CXCL8 in the absence of peptides; (*P < 0.05) as compared to control.

According to the molecular docking prediction, the crucial amino acids involved in the CXCL8–CXCR1 binding are K11, K15, and K20. We next synthesized the mutant peptides p_K11A, p_K15A, and p_K20A, in which the indicated lysine was substituted by alanine at different places to examine the effects on monocyte binding. As predicted, p_K11A was found to inhibit CXCL8-induced monocyte binding at concentration higher than 0.5 μM (Fig. 7A). However, p_K15A and p_K20A had no effects on CXCL8-induced THP-1 adhesion (Fig. 7B,C), demonstrating that amino acids K15 and K20 play crucial roles in the initial binding of CXCL8 to CXCR1.

## Discussion

In this work, a combination of molecular docking and MD simulations was used to investigate CXCL8 binding to CXCR1 embedded into a POPC lipid bilayer during the first 50 ns. Regarding CXCL8 initial binding to CXCR1, the interaction map (Fig. 1C) showed that positively charged residue of the N-loop of CXCL8 (K20) formed electrostatic interactions with negatively charged residues of the N-terminus of receptor CXCR1 (D24, E25, and D26), which is consistent with previous studies showing that the N-terminus of CXCR1 (site I) plays a crucial role in the initial recognition of the N-loop of CXCL8^12^,^14^,^15^,^16^,^18^,^19^. Recent studies have shown that monomeric CXCL8 binds to the N-terminal peptide of CXCR1 with higher affinity than does that of dimeric CXCL8, and that site I binding dominates in the initial monomer vs. dimer affinity^33^,^34^, which also support the current results. During the first 50 ns MD simulations, CXCL8 rotated slightly, with the residue K15 approaching the negatively charged residues of CXCR1 (D11, D24, and E25) through electrostatic interactions (Fig. 1D). Hydrophobic interactions between CXCL8 (Y13, P16, F17, P19, and F21) and CXCR1 (F12, F17, P21, P22, A23, L32, P29, and F172) trigger CXCL8 rotation, which allows the ELR motif of the N-terminus of CXCL8 to move closer to the extracellular loops of receptor CXCR1 (the activating step, site II binding)^12^,^19^. Calculations of the binding free energy of different CXCL8 peptides to receptor CXCR1 were also performed for advanced analysis. The three wild type peptides (p_wt14, p_wt16, and p_wt18) inhibited CXCL8-induced monocyte adhesion to HMEC-1 cells in a clearly dose-dependent manner, indicating that these peptides contain the critical binding region (Fig. 5). All positively charged peptides bound to the HMEC-1 cell surface, showing that electrostatic interactions dominate the initial binding stage. While the inhibiting effect caused by p_wt16 was slightly stronger than that of the other two peptides at a high peptide concentration (monocyte binding: p_wt16 (85%) < p_wt18 (95%) < p_wt14 (100%)), the binding free energy calculations revealed that p_wt14 was the lowest among the three peptides (Figs 3 and 5). The composition of the binding free energy of p_wt16 showed that the electrostatic interactions were weaker than those of p_wt14 due to that the positively charged residue R6 is far from the CXCR1 binding site. Compared with the solvation free energy of p_wt14, that of p_wt18 increased more because it possesses more hydrophilic residues than does p_wt14 (Fig. 3B). The difference between experiments and free energy calculations may result from that the binding free energies in theory are precisely to calculate the binding between the peptide and receptor CXCR1, whereas in cellular assays the synthesized peptide binds not only to CXCR1 but also to CXCR2 on the cell surface of HMEC-1 through the inhibition of CXCL8 binding. Bacterial endotoxin LPS, which results in overly exuberant inflammation, is one of the major causes of severe sepsis and septic shock in humans^30^,^31^. In the current study, p_wt14 not only suppresses the CXCL8-induced adhesion of monocytes to endothelial cells and monocyte trans-endothelial migration, but also modulates LPS-induced monocyte adhesion and trans-endothelial migration (Figs 5A and 6A–C). Our results also revealed that the cells treated with the peptides were able to reduce the adhesion of monocytes to LPS-stimulated endothelial cells and suppress monocyte trans-endothelial migration.

Three positively charged residues are present in CXCL8-derived peptide p_wt14 (K11, K15, and K20). To determine the key residue for p_wt14 binding to CXCR1, three mutant peptides were synthesized for the inhibition test of CXCL8 binding to HMEC-1 cells, and alanine scanning mutational analysis based binding free energy calculations were also performed for comparison. The mutant peptide p_K11A still inhibited the CXCL8 binding to the HMEC-1 cell (90%) whereas the other mutant peptides failed to exhibit any measurable inhibiting effect on monocyte binding (p_K15A: 190%; p_K20A: 175%) at a concentration of 5 μM, as predicted by the binding free energy calculations (Figs 7 and 3A). The electrostatic interactions and solvation free energy of the mutant peptide p_K15A decreased more than those of the other two mutant peptides did, indicating that K15 of CXCL8 is the key residue for peptide p_wt14 binding to CXCR1 (Fig. 3B). Moreover, SPR analysis demonstrated p_wt14 could bind to the derived peptide from the CXCR1 binding site (CXCR1p) (*K*_*D*_ = 252 μM) while p_K15A seemed to inhibit binding to CXCR1p (*K*_*D*_ = 1553 μM), which is in consistent with cell-based experiments and binding free energy calculations (Fig. 4B,D). The RUs of p_wt14 binding to CXCR1p were twofold higher than those of p_K15A, suggesting that eliminating the positive charge on the 15th residue lysine affected the binding of the peptide to CXCR1p. The *K*_*D*_ value of p_wt14 seemed to be high compared with that yielded during proper peptide ligand-receptor binding. However, this might be because the SPR measurements were performed on peptide–peptide (p_wt14-CXCR1p) binding rather than on the general ligand–antibody or antibody–cell bindings, which normally have lower *K*_*D*_ values (approximately 50 μM)^35^. Although the immobilized WT peptide p_wt14 binds to CXCR1p with a little high *K*_*D*_ value, the SPR results were able to precisely distinguish the binding between the receptor peptide (CXCR1p) and ligand peptide (p_wt14). Furthermore, the subsequent cellular assays confirmed the results. SPR measurements verified that the wild CXCL8 peptide p_wt14 binds to receptor peptide CXCR1p whereas the mutant peptide p_K15A hardly binds to CXCR1p, which is consistent with binding free energy calculations showing that as K15 is mutated to A15, the binding free energy and electrostatic interactions become less negative. In the CXCL8-induced monocyte binding assays, mutant peptides p_K15A and p_K20A could not reduce CXCL8-induced monocyte binding, and p_K11A still inhibited monocyte binding, which also agrees with previous binding free energy calculations showing that the binding free energies of p_K15A and p_K20A become less negative whereas the free energy of mutant p_K11A is similar to that of wild p_wt14.

## Conclusions

In this work, we expanded on our previous receptor model^19^ by proposing a method that combines molecular docking and binding free energy calculations to design a preferable peptide for inhibiting CXCL8 binding to CXCR1, which might be useful for the development of anti-inflammatory drugs. SPR measurements verified that p_wt14 binds to the CXCR1p derived from the binding site of the receptor, while mutant peptide p_K15A seems hardly to bind the CXCR1p. The designed peptide was also tested on cellular assays. Peptide p_wt14 reduced CXCL8-induced monocyte adhesion and transmigration, and also reduced LPS-activated monocyte binding and transmigration by suppressing CXCR1 and CXCR2 activation. The mutant peptides (p_K15A, and p_K20A) appeared not to inhibit CXCL8-induced monocyte binding, indicating that residues K15 and K20 of CXCL8 may be the key residues responsible for p_wt14 blocking CXCL8 binding to CXCR1, as suggested by the binding free energy calculations. The theoretical computational simulations agree with our experimental results, indicating that *in silico* investigation is a powerful tool in the development of anti-inflammatory peptides.

## Methods

### Construction of the full-length CXCR1

Due to the highly flexible N- and C-terminal parts of rhodopsin-like GPCRs, to date, the most resolved structures, such as CXCR1 (PDB code: 2LNL), β_2_AR (PDB code: 2RH1), A_2_AR (PDB code: 3EML) and CXCR4 (PDB code: 3ODU) still lacked N- or C-terminal part whereas only the bovine rhodopsin structure was resolved including N- and C-terminal domains (PDB code: 1U19). Bovine rhodopsin structure was therefore used as a template for homology modeling the receptor CXCR1^36^,^37^. Based on our pervious study^19^, full-length CXCR1 was constructed by combining the NMR experiments from Opella, S. J. *et al.*^38^ (PDB code: 2LNL) and the homology modeling results. Homologous models of the N-terminus (residues 2~28) and the C-terminus (residues 325~347) of CXCR1 were created by using the Phyre2 web server (http://www.sbg.bio.ic.ac.uk/phyre2/)^39^. The two modeled terminal parts and CXCR1_29–324_ were then merged together to form a full-length CXCR1 structure using the MOE software package (Molecular Operating Environment, http://www.chemcomp.com). The full-length CXCR1 structure was following embedded into a fully hydrated POPC lipid bilayer (16:0−18:1 diester PC, 1-palmitory-2-oleoyl-sn-glycero-3-phospho-chloine) for 100 ns MD simulations to derive a relaxation of the conformation.

### Molecular Docking

The initial favorable sites for CXCL8 binding to the receptor CXCR1 were determined by using the “dock proteins protocol” (ZDOCK) from Discovery Studio 3.5 (Accelrys Inc., San Diego, CA). Previous studies have demonstrated that the N-terminal domain of receptor CXCR1 plays a crucial role in the ligand CXCL8 binding^12^,^14^,^15^,^16^,^20^,^32^. A general two-sites model with multi-steps also revealed that chemokine-receptor binding involves two interactions: between the N-loop of CXCL8 and the N-terminus of CXCR1 (site I) and between the N-terminal part of CXCL8 and the extracellular loops of CXCR1 (site II)^14^,^19^. Based on these studies, the molecular docking was set to block the transmembrane domain and all the intracellular residues but to allow the N-terminal domain, extracellular loops for ligand binding. Residues except for the extracellular domains of CXCR1 (residues 38~97, 116~174, 206~262, and 286~347) were blocked to reduce the computing cost and enhance the docking accuracy. Detailed docking procedures are also described in Supplementary Information.

### Molecular Dynamics (MD) simulations

All complexes were inserted into a POPC lipid bilayer system (2 × 144 lipids) by removing overlapping lipids and water molecules. For the system of CXCL8 (PDB code: 3IL8) in complex with CXCR1, after energy minimization, 61 Na^+^ and 75 CL^−^ ions (generating a 100 mM NaCL solution) were added to neutralize the whole system. Finally, the patches consisted of the complex structure (4305 atoms), 238 POPC lipids, and 20987 SPC-water molecules including 61 Na^+^ and 75 CL^−^ ions (79778 atoms in total). All complex simulations were run for 50 ns. All MD simulations were carried out with GROMACS-4.5.5 using Gromos96 (ffG45a3) force field with an integration step size of 2 fs. Detailed MD simulation settings are described in Supplementary Information.

A principal component analysis (PCA) over the backbone atoms of all frames of the last 30 ns of each of the complex system was also performed. A structure was calculated averaging over the first few eigenvectors. PCA was accomplished using the program g_covar from the GROMACS-4.5.5 package. Rotational and translational motions were removed by fitting the peptide structure of each time frame to the starting structure.

### MM/PBSA binding free energy calculations

To determine the most stable complexes predicted by the molecular docking, the binding free energy (Δ*G*_*bind*_) for each complex was estimated by using the MM/PBSA approach exploited previously based on the snapshots extracted from the single trajectory of the complex (single trajectory method)^40^,^41^. Compared with the individual trajectory method, which requires three separate MD simulations on the three system components (the receptor, the ligand, and the complex) to calculate the binding free energy, the single trajectory method is much faster and requires less sampling^42^,^43^. In this case, the receptor and the ligand are assumed to behave similarly in the binding, which is reasonable and adopted for our simulations. Accordingly, the binding free energy of a ligand to a receptor in a solution is defined as the following equation^44^,^45^:


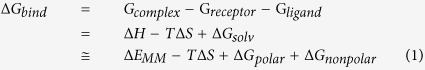


where Δ*E*_*MM*_ is the change of the gas-phase molecular mechanics (MM) energy, defined as:


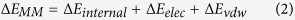


where Δ*E*_*internal*_ indicates bond, angle, and torsional angle energies, Δ*E*_*elec*_ and Δ*E*_*vdw*_ denote the electrostatic and van der Waals energies, respectively. In Eq. (1), Δ*G*_*solv*_ is the sum of electrostatic solvation energy (polar contribution), Δ*G*_*polar*_, and the nonelectrostatic solvation energy (nonpolar contribution), Δ*G*_*nonpolar*_. In this work, Δ*G*_*polar*_, and Δ*G*_*nonpolar*_ were calculated with APBS (Adaptive Poisson-Boltzmann Solver program)^46^. The polar contribution Δ*G*_*polar*_ refers to the energy required to transfer the solute from a continuum medium with a low dielectric constant (ε = 1) to a continuum medium with the dielectric constant of water (ε = 80). Δ*G*_*polar*_ was calculated using the non-linearized Poisson-Boltzmann equation. The grid spacing was automatically set to an upper limit of 0.5 Å. The temperature was set to 296 K, and the salt concentration was 0.15 M. Δ*G*_*nonpolar*_ was considered proportional to the solvent accessible surface area (SASA):


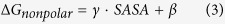


where γ = 0.0227 kJmol^−1 ^Å^−2^ and β = 0 kJmol^−1^ 47. The dielectric boundary was defined using a probe of radius 1.4 Å. Moreover, −TΔ*S* is the change of the conformational entropy upon ligand binding, and was not calculated herein due to the expensive computing cost and poor prediction accuracy^41^,^42^. Finally, a total of 300 snapshots extracted from the last 30 ns stable MD trajectory per system were used for calculating all energy terms. The binding free energy calculating tool in Gromacs 4.5.5 (GMXAPBS tools) is supported from Musco group^45^.

### Peptide synthesis

Based on the *in silico* analysis of this study, peptides and their mutants were designed for experimental confirmation. These peptides were chemically synthesized by GeneMark (GMbiolab Co., Ltd., Taiwan) with a solid phase methodology. Peptides used in this study were summarized in Table 1.

### Surface Plasmon Resonance (SPR) Measurements

Two peptides (p_wt14 and p_K15A) designed based on ZDOCK and binding free energy calculations were tested experimentally using SPR to determine whether they can bind to receptor peptide CXCR1p. The SPR measurements were performed using a Biacore T200 workstation (GE Healthcare, USA) and synthesized peptides (wild-type, mutant peptides derived from CXCL8 and CXCR1). 10 μM of p_wt14 and p_K15A were diluted into 10 mM sodium acetate buffer at pH 5.0 and immobilized onto a CM5 sensor chip using amine coupling (EDC-NHS) for 6 min at a flow speed of 5 μl/min. Approximately 1600 response units (RU) of p_wt14 (or p_K15A) were immobilized on the chip. CXCR1p binding to p_wt14 was used as a positive control. Three single cycle kinetic studies were performed to compare the binding of CXCR1p to the wild p_wt14 and mutant p_K15A peptides. In both studies, data on the kinetics and affinity of ligand-receptor binding were obtained by flowing seven concentrations of the CXCR1p (0 μM, 15 μM, 30 μM, 120 μM, 240 μM, 360 μM and 480 μM) over the chip sequentially at a flow rate of 50 μl/min for 2 min. The response was measured at two time points during 0 and 120 seconds to obtain information on the rate of CXCR1p association with and dissociation from p_wt14 (or p_K15A). Equilibrium binding curves were generated and fitted using a monovalent binding model for each peptide to determine the K_D_ of the binding of CXCL8 peptides (p_wt14 and p_K15A) with receptor peptide CXCR1p.

### Cell Culture

Human micro-vascular endothelial cell-1 (HMEC-1, from American Type Culture Collection Manassas, USA) was maintained in MCDB 131 medium (Sigma, USA) with L-glutamine and fetal bovine serum (Sigma, USA) to a final concentration of 15%. The medium was supplemented with 500 units/ml penicillin (Biowest, USA), 500 μg/ml streptomycin (Biowest, USA), 10 ng/ml Epidermal Growth Factor (ProSpec, USA) and 1□g/ml hydrocortisone (Sigma, USA). The THP-1 cells were maintained in the RPMI-1640 medium (Invitrogen, USA) with 10% FBS.

### Cell Adhesion Assay

For the assay of THP-1 cell adhesion to HMEC-1 monolayers, confluent HMEC-1 cell monolayers cultured on 24-well plates were pretreated with peptides for one hour and activated with 25 ng/ml LPS (Sigma, USA) or full length CXCL8 (ProSpec, USA) for 18 hours. THP-1 monocytes were suspended in RPMI 1640 and prelabeled with 5 mM Calcein-AM (Invitrogen, USA) for 30 minutes. Before the THP-1 cells were added to HMEC-1, the monolayers were washed twice to remove LPS or CXCL8. Fluorescence labeled monocytes (3 × 10^5 ^cells/well) were then added to HMEC-1 and incubated for 30 minutes at 37 °C. Non-adhered monocytes were removed by five times of gentle wash with Hank’s balanced salt solution. The images of adhered THP-1 cells were photographed (five images per well) under fluorescent microscopy and the number of attached monocytes was counted with the software AlphaImager 2200 (Alpha Innotech, USA).

### Transendothelial Migration

Migration of THP-1 cells across HMEC-1 was performed in a Transwell system (BD Bioscences, USA). Briefly, HMEC-1 cells cultured on transwell (polycarbonate membranes with 8 μm pore sizes) filters were pretreated with peptides for one hour and then stimulated with 25 ng/ml LPS or CXCL8 for 18 hours. THP-1 monocytes (2 × 10^5^ cells/well) were added to the upper chamber of transwell inserts containing 50 μl RPMI 1640. LPS (25 ng/ml), CXCL8 peptide (p_wt14) (0.5 and 5 mM), or both combinations were added to the lower chamber to initiate transmigration. After four hours of incubation at 37 °C under 5% CO_2_, cells that had transmigrated to the lower chamber were harvested and counted using microscopy. All experiments were measured in at least three independent studies, and the data represent the number of transmigrated THP-1 cells.

### Cell Viability Assay

To evaluate if the peptide p_wt14 is toxic to the HMEC-1 under resting conditions, 2 × 10^4^ cells were dispensed into each well of 96-well micro-plates and incubated for 2 hours for adhesion. After that, the medium containing peptide p_wt14 at various concentrations was added. After 24 and 48 hours of incubation, cells were washed with Hank’s balanced salt solution and the viabilities of HMEC-1were determined by WST-1 assay kit (Roche, USA). Each experiment was performed in quadruplicate and repeated at least three times.

## Additional Information

**How to cite this article**: Jiang, S.-J. *et al.* Peptides derived from CXCL8 based on *in silico* analysis inhibit CXCL8 interactions with its receptor CXCR1. *Sci. Rep.* **5**, 18638; doi: 10.1038/srep18638 (2015).

## Supplementary Material

Supplementary Information

## Figures and Tables

**Table 1:** Summary of all synthesized peptides.

No.	Type	Name	Peptide sequence
1	Wild	p_wt14	^8^QCIKTYSKPFHPKF^21^
2	Wild	p_wt16	^6^RCQCIKTYSKPFHPKF^21^
3	Wild	p_wt18	^6^RCQCIKTYSKPFHPKFIK^23^
4	Mutant	p_K11A	^8^QCIATYSKPFHPKF^21^
5	Mutant	p_K15A	^8^QCIKTYSAPFHPKF^21^
6	Mutant	p_K20A	^8^QCIKTYSKPFHPAF^21^
7	Wild	CXCR1p	^11^DFDDLNFTGMPPADEDYS^28^

Wild type (p_wt14, p_wt16, p_wt18) and mutant (p_K11A, p_K15A, p_K20A) peptides are derived from ligand CXCL8 while CXCR1p is derived from receptor CXCR1.
