# Supplementary material for: Peptides derived from CXCL8 based on in silico analysis inhibit CXCL8 interactions with its receptor CXCR1
Source: Sci Rep. 2015 Dec 22;5:18638. doi: 10.1038/srep18638 (PMC4686899; doi:10.1038/srep18638)
Supplement: Supplementary Information [file srep18638-s1.doc]

**Supplementary Information**

**Peptides derived from CXCL8 based on *in silico* analysis inhibit CXCL8 interactions with its receptor CXCR1**

Shinn-Jong Jiang^1^, Je-Wen Liou^1,2^, Chun-Chun Chang^2,3^, Yi Chung^4^, Lee-Fong Lin^4^ and Hao-Jen Hsu^4*^

^1^Department of Biochemistry, School of Medicine, Tzu Chi University, Hualien 97004, Taiwan, ^2^Institute of Medical Sciences, Tzu Chi University, Hualien 97004, Taiwan, ^3^Department of Laboratory Medicine, Tzu Chi Medical Center, Hualien 97004, Taiwan, ^4^Department of Life Sciences, Tzu Chi University, Hualien 97004, Taiwan.

**
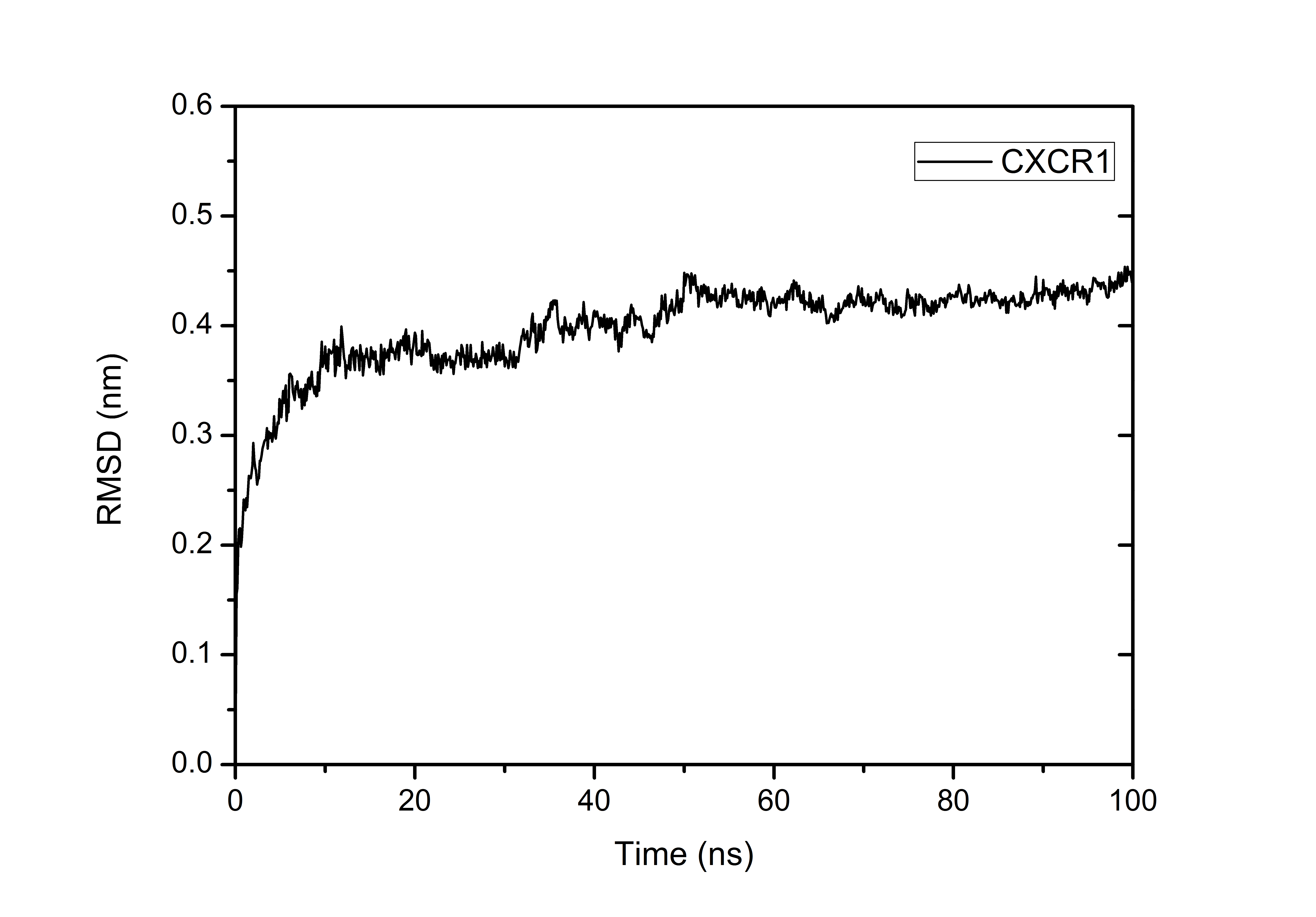
**

**Figure S1: RMSD values of modeled full-length CXCR1 during MD simulations.** Plot of the RMSD for the backbone atoms of CXCR1 embedded into POPC lipid bilayers throughout 100 ns MD trajectory.

**
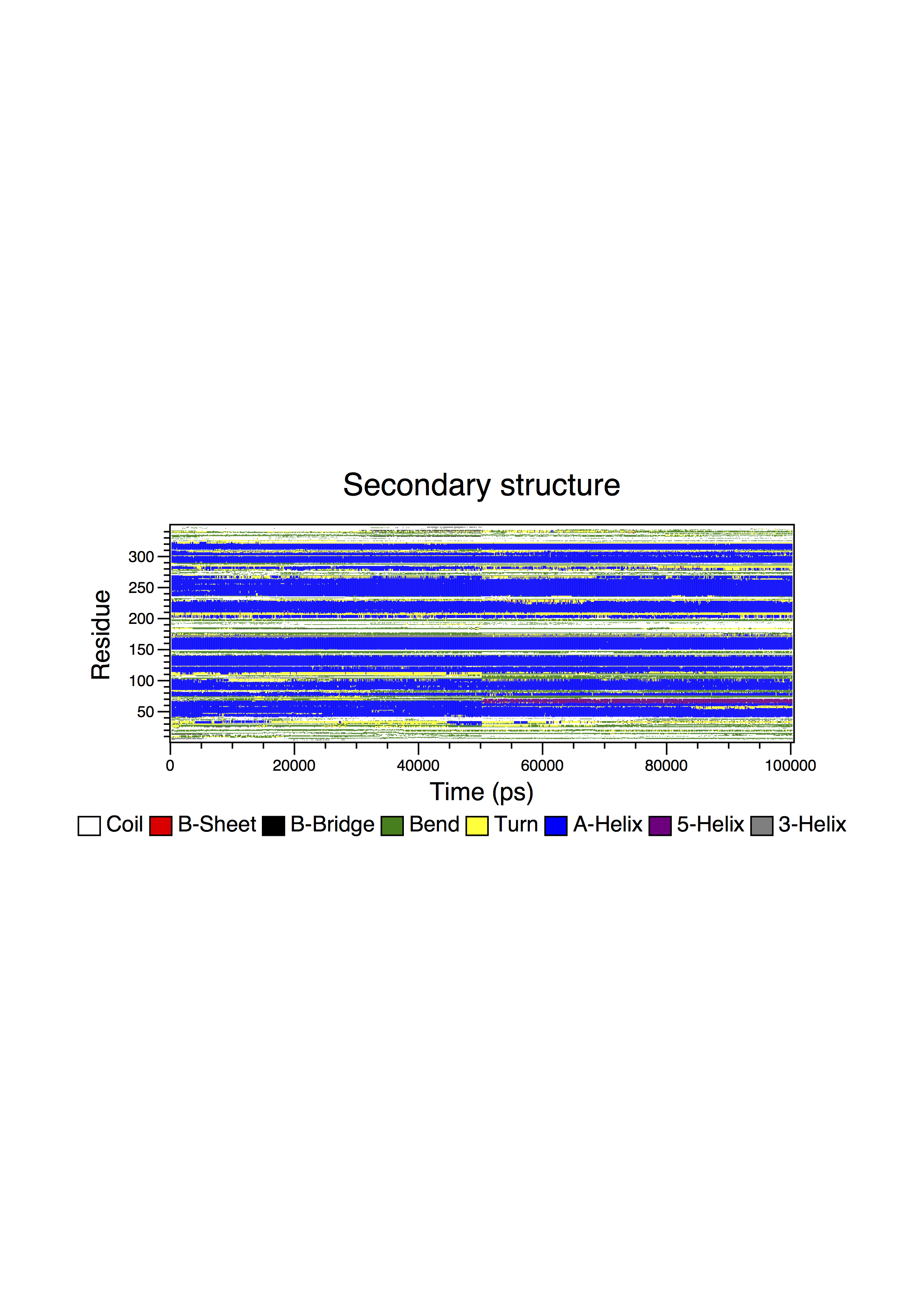
**

**Figure S2: The DSSP plot assign secondary structure information to the residues of receptor CXCR1.** The change of secondary structure elements of modeled receptor CXCR1 during the 100 ns simulations indicates that N-terminal part (residues 1~35), and extracellular parts (EC1: residues 102~108, EC2: residues 173~198, and EC3: residues 277~284) remain random coil and loop forms during the simulations.

**
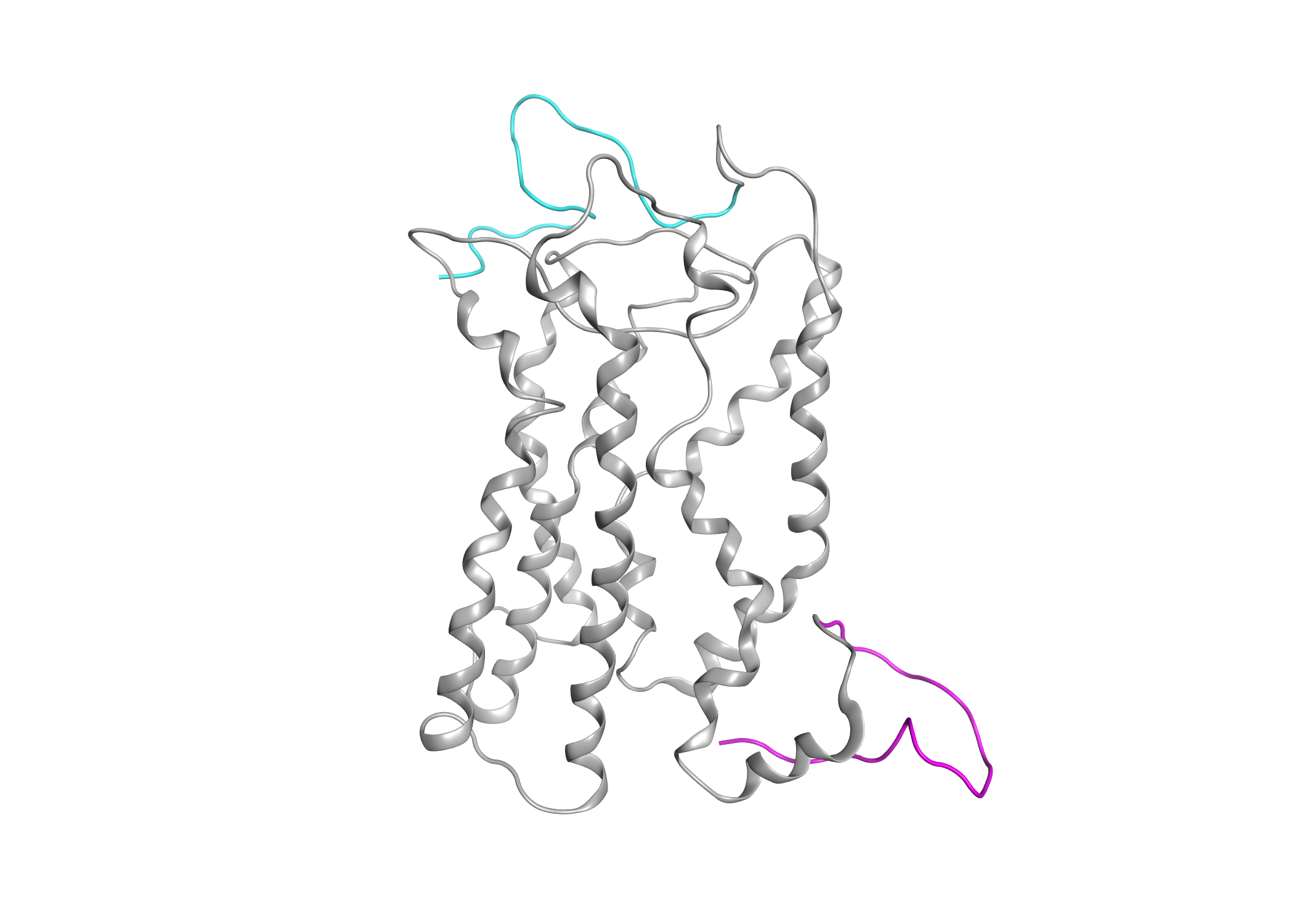
**

**Figure S3: Average structure of full-length CXCR1.** Ribbon representations of the average modeled full-length receptor CXCR1 (residues 2~347). The average structure is obtained based on PCA of the covariance matrix resulting from the last 30 ns MD trajectory. The CXCR1 is composed of the structure from the NMR experiment (residues 29~324, gray color), the N-terminal (residues 2~28, light blue color) and C-terminal (residues 325~347, pink color) domains from homology modeling results.

A


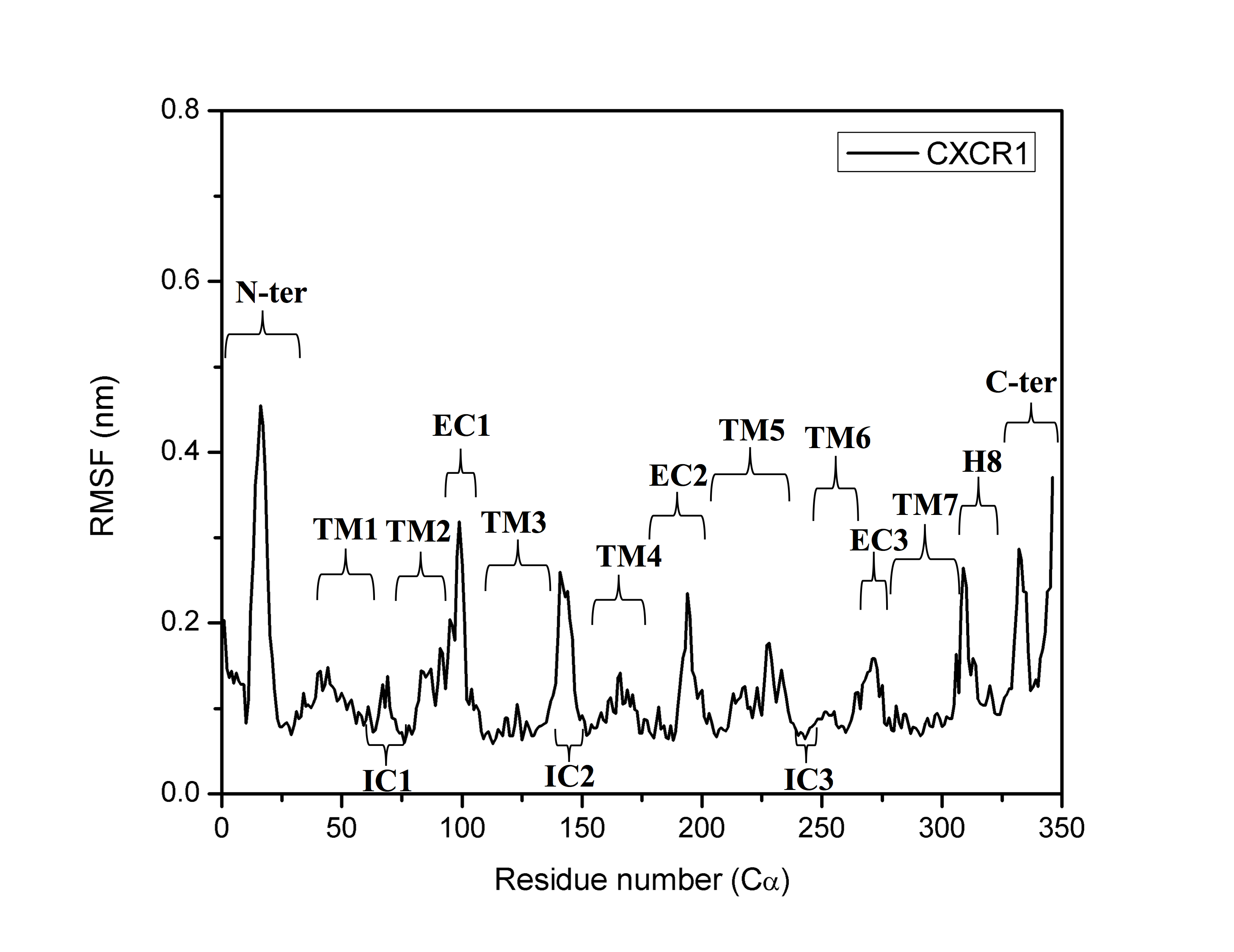


B


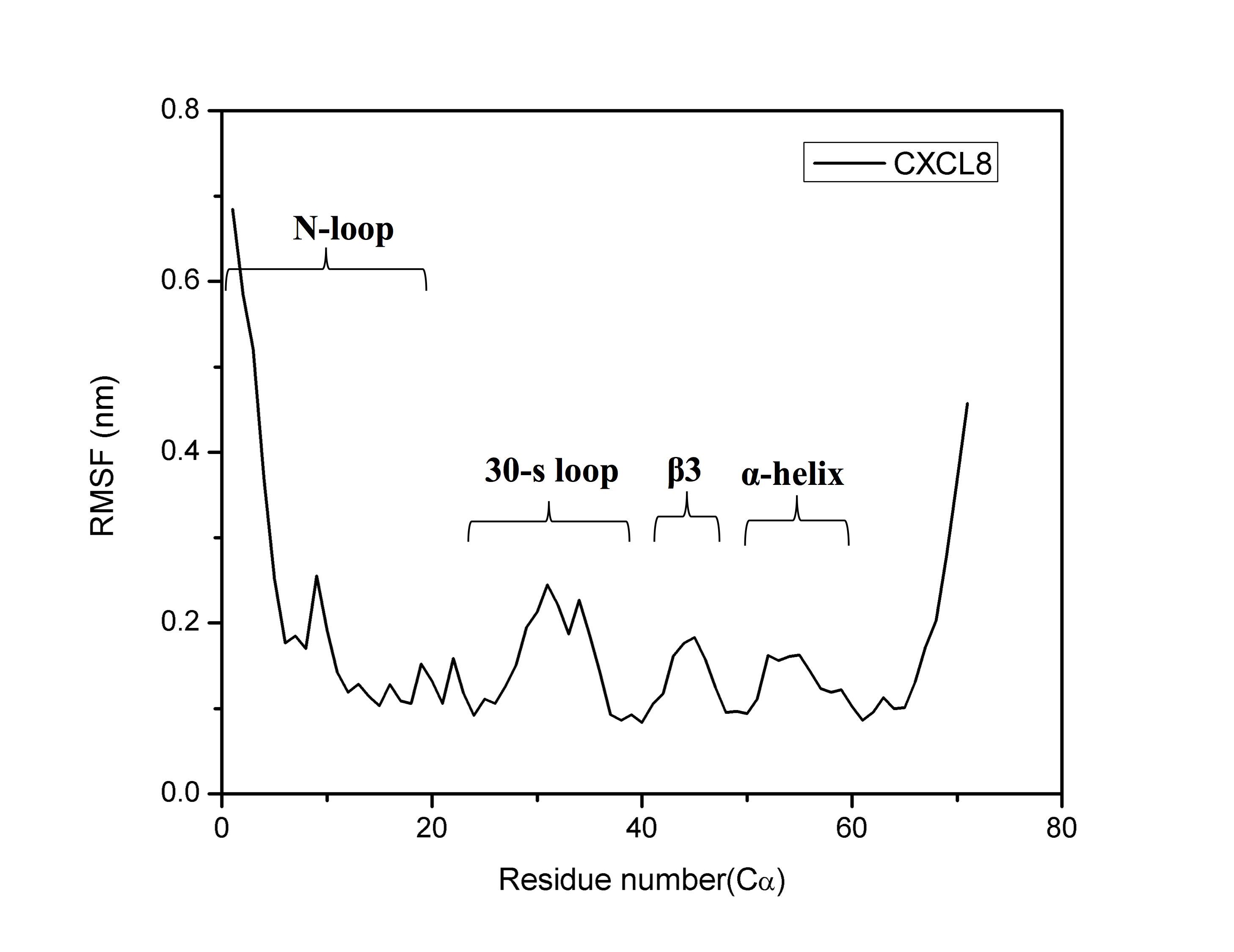


**Figure S4: Plots of RMSF values for the Cα atom of complex structure.** The location of the terminus (N-ter, C-ter), TM helices, IC-loops, and EC-loops are marked in the figure. (A) CXCR1 (B) CXCL8.


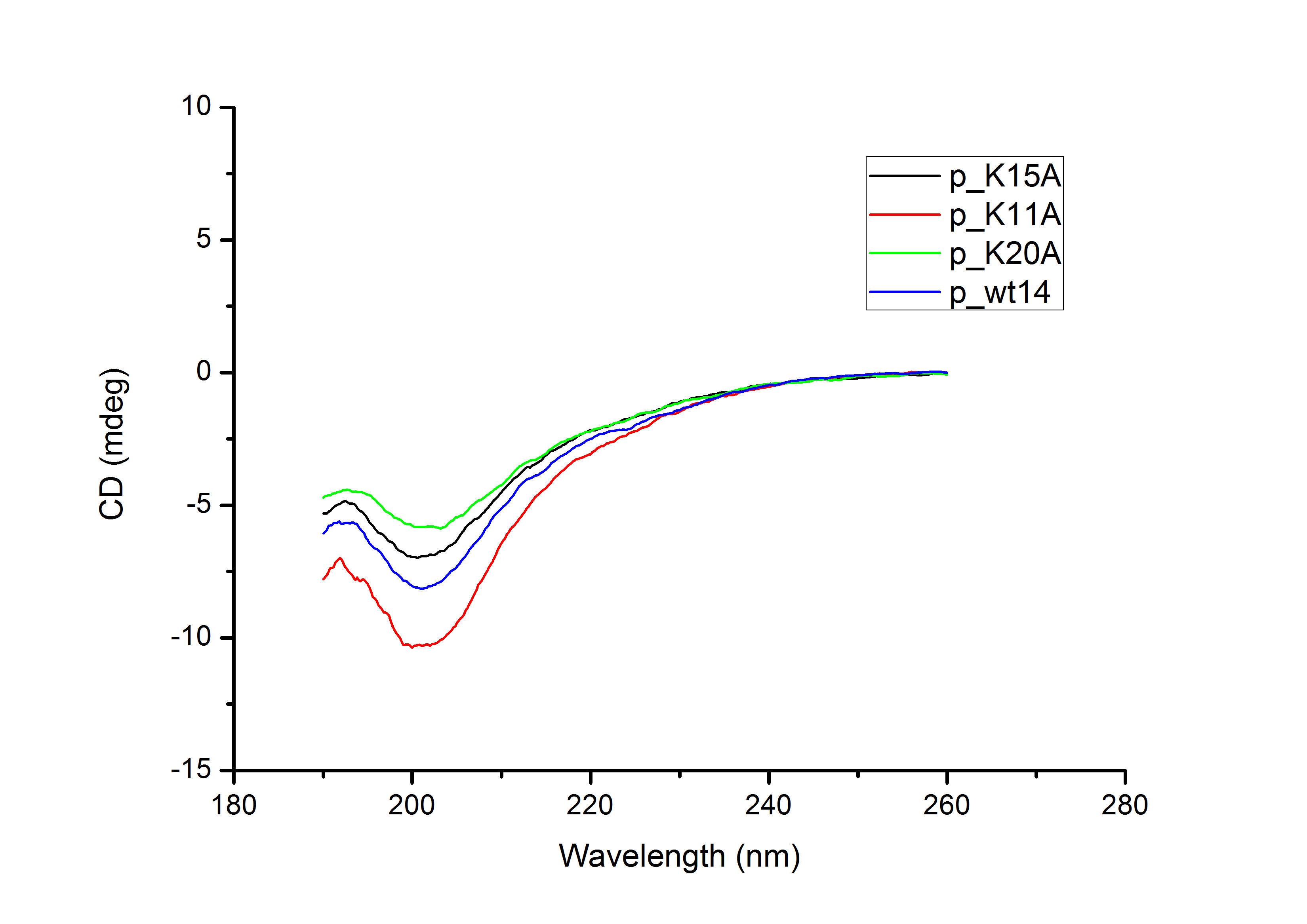


**Figure S5: CD spectra of various peptides in water solution.** Blue line: wild CXCL8 peptide with 14 amino acids (p_wt14); Red line: mutant CXCL8 peptide with K11A; Black line: mutant CXCL8 peptide with K15A; Green line: mutant CXCL8 peptide with K20A. Spectra were recorded from 190 to 260 nm as an average of eight scans and smoothed to obtain the final data. All the CD spectra showed the random coil conformations.


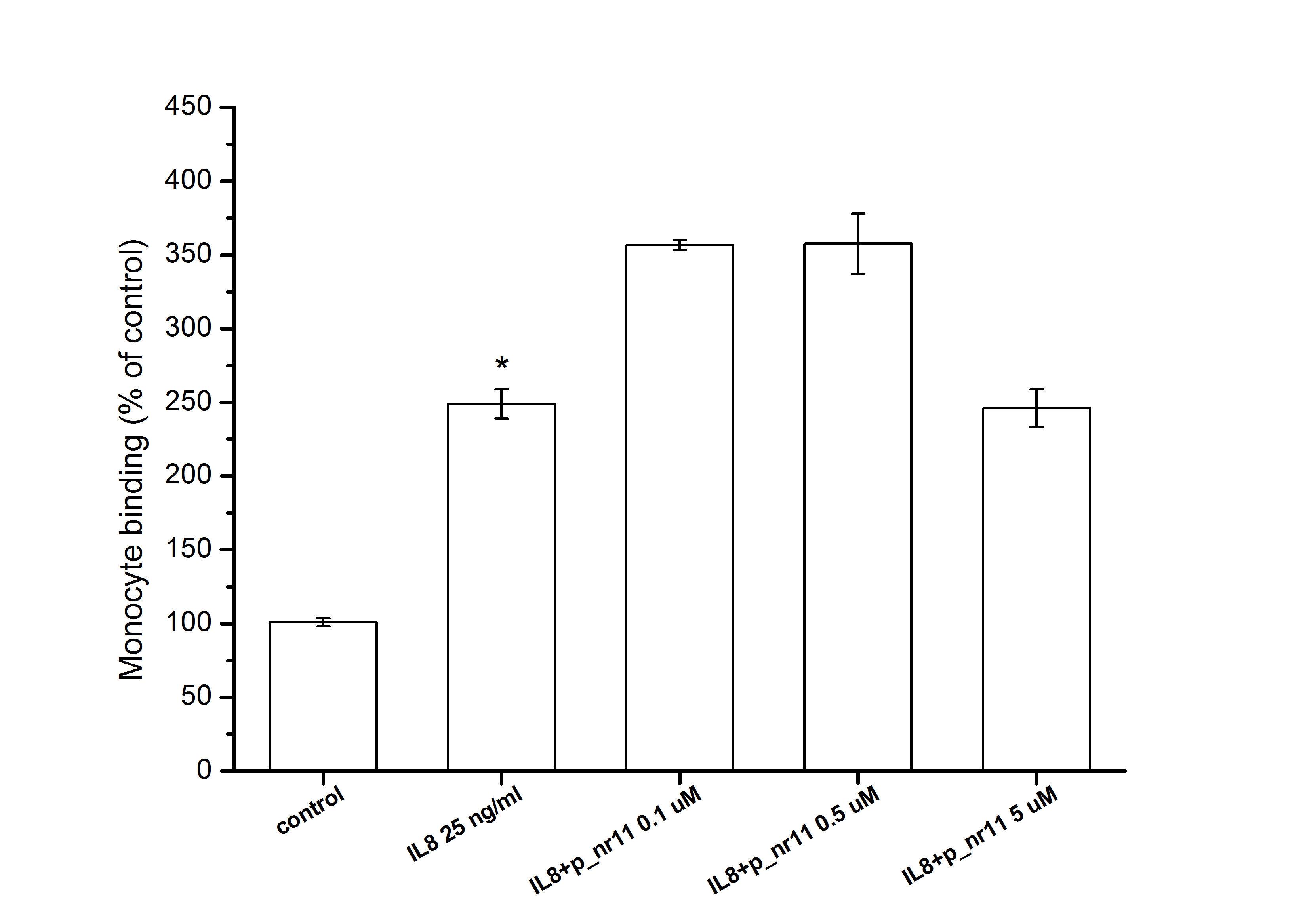


**Figure S6: Effect of the non-related peptide on the inhibition of CXCL8-induced monocyte adhesion to HMEC-1.** HMEC-1 was pretreated with various concentrations of the nonrelated peptide for one hour, and then stimulated with 25 ng/ml CXCL8 for 18 hours. Adhesion of fluorescent THP-1 cells was photographed by fluorescent microscopy and calculated. “Control” means that only the culture medium (without peptides) is incubated with cells. Values are mean ± SD from three independent experiments. (* *P* < 0.05) as compared to the control.

Table

Table S1: Calculated binding free energies and individual energy components for the various systems (kcal/mol)

| *Systems* | *ΔG_bind_* | *ΔE_elec_* | *ΔE_vdw_* | *ΔG_solv_* | *ΔG_polar_* | *ΔG_nonpolar_* |
| --- | --- | --- | --- | --- | --- | --- |
| CXCL8  (IL-8) | -255.10  ±5.81 | -337.99  ±16.59 | -123.03  ±3.92 | 205.92  ±3.43 | 220.00  ±6.47 | -14.09  ±0.39 |
| p_wt14 | -134.49  ±4.22 | -212.48  ±8.73 | -59.29  ±2.36 | 137.27  ±1.73 | 144.53  ±3.25 | -7.25  ±0.22 |
| p _wt16 | -96.06  ±5.44 | -176.38  ±10.34 | -66.67  ±3.44 | 146.99  ±2.53 | 155.00  ±4.78 | -8.02  ±0.29 |
| p _wt18 | -66.24  ±5.87 | -173.77  ±9.28 | -89.12  ±2.80 | 196.66  ±1.76 | 207.63  ±3.32 | -10.97  ±0.20 |
| p_K11A | -122.06  ±3.66 | -191.20  ±6.62 | -59.08  ±2.35 | 128.22  ±1.34 | 135.47  ±2.46 | -7.25  ±0.22 |
| p_K15A | -68.80  ±3.72 | -92.70  ±6.64 | -52.61  ±2.06 | 76.50  ±1.34 | 83.22  ±2.44 | -6.72  ±0.23 |
| p_K20A | -97.10  ±3.82 | -172.32  ±6.78 | -54.74  ±2.28 | 129.96  ±1.37 | 136.87  ±2.50 | -6.91  ±0.23 |

Table S2: Summary of IC50 values for wild type CXCL8 peptides (p_wt14, p_wt16, and p_wt18) used to inhibit CXCL8 induced monocyte adhesion to HMEC-1 cells.

| Peptides | p_wt14 | p_wt16 | p_wt18 |
| --- | --- | --- | --- |
| IC50 (μM) | 0.18 | 0.05 | 0.07 |

IC50 values are obtained by fitting the inhibition-concentration curves shown in Figure 5.

**Supplementary methods**

***Molecular Docking***

The ZDOCK protocol was used to conduct the rigid-body docking of two protein structures as well as clustering the poses according to the ligand position using a Fast Fourier Transformation (FFT) to perform an exhaustive six-dimensional search in the translational and rotational space between the two molecules. The rotational search sampling grid is used as a 6^o^ grid which samples a total of 54000 docked poses per system. ZDOCK searches conformational space by rotating the ligand around its geometric center with the receptor kept fixed in space. The ZRANK function, as part of the ZDOCK protocol, was used to re-rank the docked poses. The obtained complex configurations were ranked based on a scoring function of a linear-weighted sum of van der Waals energies, electrostatics and desolvation energies. Higher scores obtained from the ZDOCK program mean that, the complex structures are of better quality. The poses generated from ZDOCK were clustered into a maximum of 50 groups. The RDOCK protocol can be used subsequently for further refinement of the dozens of poses with higher ZDOCK scores, using a CHARMm-based energy minimization scheme for the optimization of intermolecular interactions. Scoring is based on a CHARMm electrostatic energy term and a desolvation energy term.38 The RDOCK scores are defined as the summation of the electrostatic energy from the predicted complex after minimization and the desolvation energy from the complex. The structure with the lowest RDOCK scores is selected for further MD simulations. ***Molecular Dynamics (MD) simulations***

The MD simulation protocol was as followed, after energy minimization and equilibration, 50 ns production runs were carried out without any constraint on the complex structure. The simulations were conducted in the *NPT* ensemble employing the velocity-rescaling thermostat at constant temperature 310 K, and 1 bar. The temperature of the complex protein, lipids and the solvent were separately coupled with a coupling time of 0.1 ps. Semi-isotropic pressure coupling was applied with a coupling time of 0.1 ps and a compressibility of 4.5 x10^−5^ bar^−1^ for the xy-plane as well as for the z-direction. Long-range electrostatics is calculated using the particle-mesh Ewald (PME) summation algorithm with grid dimensions of 0.12 nm and interpolation order 4. Lennard-Jones and short-range Coulomb interactions were cut off at 1.4 and 1.0 nm, respectively. The following equilibration protocol was used: (i) the temperature was gradually increased from 100 K to 200 K and 310 K. The system was run for 500 ps for the each temperature. During these simulations the complex structure remained fully restraint (k = 1000 kJ mol^-1^ nm^-2^). (ii) At 310 K the restraints kept on the complex structure via the force constant k, were released in 3 steps from k = 500 kJ mol^-1^nm^-2^ to k = 250 kJ mol^-1^nm^-2^, and finally k = 100 kJ mol^-1^nm^-2^. Each step was run for 2.0 ns.
